# Connecting phenotype to genotype: PheWAS-inspired analysis of autism spectrum disorder

**DOI:** 10.3389/fnhum.2022.960991

**Published:** 2022-10-12

**Authors:** John Matta, Daniel Dobrino, Dacosta Yeboah, Swade Howard, Yasser EL-Manzalawy, Tayo Obafemi-Ajayi

**Affiliations:** ^1^Department of Computer Science, Southern Illinois University Edwardsville, Edwardsville, IL, United States; ^2^Department of Computer Science, Missouri State University, Springfield, MO, United States; ^3^Department of Translational Data Science and Informatics, Geisinger, Danville, PA, United States; ^4^Engineering Program, Missouri State University, Springfield, MO, United States

**Keywords:** autism spectrum disorders, graph-based clustering, PheWAS, genotypes, kNN graphs

## Abstract

Autism Spectrum Disorder (ASD) is extremely heterogeneous clinically and genetically. There is a pressing need for a better understanding of the heterogeneity of ASD based on scientifically rigorous approaches centered on systematic evaluation of the clinical and research utility of both phenotype and genotype markers. This paper presents a holistic PheWAS-inspired method to identify meaningful associations between ASD phenotypes and genotypes. We generate two types of phenotype-phenotype (p-p) graphs: a direct graph that utilizes only phenotype data, and an indirect graph that incorporates genotype as well as phenotype data. We introduce a novel methodology for fusing the direct and indirect p-p networks in which the genotype data is incorporated into the phenotype data in varying degrees. The hypothesis is that the heterogeneity of ASD can be distinguished by clustering the p-p graph. The obtained graphs are clustered using network-oriented clustering techniques, and results are evaluated. The most promising clusterings are subsequently analyzed for biological and domain-based relevance. Clusters obtained delineated different aspects of ASD, including differentiating ASD-specific symptoms, cognitive, adaptive, language and communication functions, and behavioral problems. Some of the important genes associated with the clusters have previous known associations to ASD. We found that clusters based on integrated genetic and phenotype data were more effective at identifying relevant genes than clusters constructed from phenotype information alone. These genes included five with suggestive evidence of ASD association and one known to be a strong candidate.

## 1. Introduction

Autism Spectrum Disorder (ASD) is a childhood neurodevelopmental disorder diagnosed on the basis of behavioral assessments of social, communicative, and repetitive symptoms. Although ASD is behaviorally distinctive and reliably identified by experienced clinicians, it is clinically and genetically an extremely heterogeneous disorder which is assumed to reflect multiple etiologic origins (Tammimies et al., 2015). The heterogeneity in ASD is multidimensional and complex, including variability in phenotype as well as clinical, physiological, and pathological parameters. ASD is associated with a wide range of cognitive and behavioral abnormalities (Chang et al., 2015). For a set of patients with the same ASD diagnosis, the clinical phenotypes could be extremely different, and could also vary significantly in terms of severity (Lovato et al., 2019). There is a pressing need for a better understanding of the heterogeneity of autism based on scientifically rigorous approaches centered on systematic evaluation of the clinical and research utility of both phenotype and genotype markers (Georgiades et al., 2013). A key challenge to finding effective solutions lies in connecting the genetic and etiological data to behavioral and phenotypic data (Miles, 2011) which is further compounded by the enormous genetic complexity of ASD-related conditions. Though there is abundant evidence of the role of genetics in the disorder, our understanding of the specific underlying genes is still limited. It is estimated that a thousand or more genes may be implicated with ASD, but only about a hundred are considered strongly linked (Brueggeman et al., 2020). The gene functional network analysis conducted in Chang et al. (2015) attempts to elucidate the genetic variations associated with autism and other psychiatric disorders by incorporating relevant phenotype data. The results suggest that its pathophysiological heterogeneity is matched by the diversity of genetic and functional causes associated with the disorder. The elucidation of the genetics behind ASD has been contributing significantly to phenotype elucidation (Lovato et al., 2019; Narita et al., 2020). ASD genotype-phenotype correlations are important to identify phenotypic subtypes and for early diagnosis and clinical management (Wu et al., 2020). Since ASD manifests significant differences across probands, to further understand how functional properties of implicated genes affect phenotypic characteristics of the disease, it might be helpful to identify more homogeneous groups by linking the phenotype to the genotype. However, ASD's highly complex genetic architecture makes it difficult to map its heterogeneity with specific phenotype-genotype relationships. Approaches that are more inclusive in relation to both are needed for further progress in mapping genotype-phenotype relationships in ASD (Binder, 2021).

Phenome-wide association studies (PheWAS) (Denny et al., 2010) have contributed substantially to uncovering etiological links between genes and diseases, as these studies can provide a more complete understanding of the complex relationships among genetic architecture and the functions of biological systems (Tyler et al., 2016; Verma et al., 2019). A PheWAS study employs regression techniques on genetic information [single nucleotide polymorphisms (SNPs)] of a given sample population of probands to derive an association between observed phenotypes and SNPs. The traditional PheWAS output yields a plot of the statistical significance power of association of multiple diseases (for each one) to a single SNP. The PheWAS concept is very effective as even studies with small sample sizes have been able to validate previous studies and enhance single SNP results with pleiotropic relationships (Hebbring et al., 2013). In Matta et al. (2021), we presented a simple PheWAS model to identify SNP associations among ASD phenotypes. Our hypothesis was that by viewing a heterogeneous disorder, such as ASD, as an aggregate of multiple subtypes, we could derive a PheWAS model to identify novel genetic and cross-phenotype associations. In our preliminary work, a multi-trait mixed regression model was applied to both SNP and phenotype matrices to emulate the PheWAS model. A phenotype-phenotype (p-p) network was obtained by linking phenotypes that have common associated SNPs. The network was subsequently clustered using the Louvain method to yield a set of phenotype-based clusters which were analyzed to reveal their associated genes. Note, this approach differs from the PheWAS model in Gutiérrez-Sacristán et al. (2021) which is based on sex differences in ASD and uses certain clinical features to identify potential disease subgroups.

**Figure 1:**
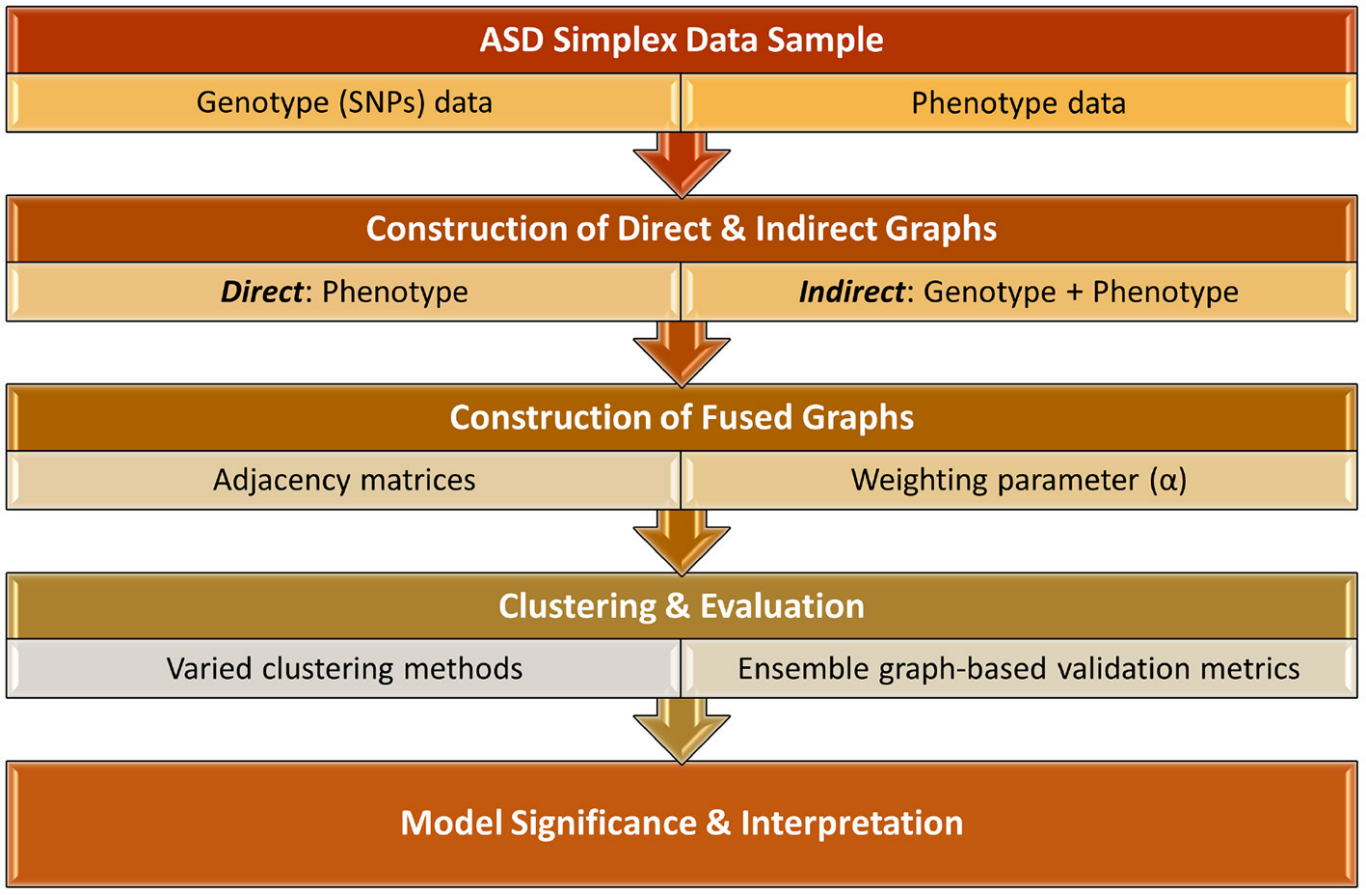
Overall framework of identifying key associations between ASD phenotype and genotype biomarkers.

In this study, we investigate a more holistic PheWAS-inspired method to identify meaningful associations between ASD phenotypes and genotypes. This work, as shown in Figure 1, significantly extends the preliminary results presented in Matta et al. (2021). We examine multiple methodologies for implementing a p-p network, creating both direct networks using only phenotype data as well as indirect networks that incorporate SNP data. These two types of networks are fused using a novel algorithm that varies the influence of direct and indirect factors. The resulting networks are clustered using a variety of network-based clustering techniques. The cluster variations among the results, based on the weighted combinations, are examined using heatmaps. To determine the optimal clustering result, we utilize an ensemble of six graph-based validation metrics. We extract the underlying genes of the highest ranking clustering results and conduct a rigorous biological analysis of these phenotype clusters and associated genes to examine their potential clinical significance. Presenting a new classification system for heterogeneous ASD instances is beyond the scope of this paper. However, to the best of our knowledge, most prior ASD clustering work (Bruining et al., 2010; Matta et al., 2018b) directly clusters the samples in an attempt to uncover more homogeneous subgroups of the sample population. In this research, we demonstrate an empirical framework for using genotype and phenotype information to cluster ASD samples in a non-traditional way. With this approach, we are able to provide empirical evidence that strengthens the case for including genotype markers in ASD diagnosis and give additional evidence for the association of several previously-known ASD-related SNPs.

## 2. Methods

### 2.1. Data

The ASD data analyzed in this work are drawn from the Simons Simplex Collection (SSC) (Fischbach and Lord, 2010), supported by the Simons Foundation for Autism Research Initiative (SFARI). (This research has been conducted under the guidelines and approval of the Institutional Review Boards at both Southern Illinois University Edwardsville and Missouri State University). The term *simplex* indicates a family in which one child is affected with an autism spectrum disorder, and the parents and siblings are not affected. Because the SSC data are simplex, none of the samples are genetically related. The SSC dataset spans over 2,759 simplex probands who have been diagnosed with ASD using the Autism Diagnostic Interview−Revised (ADI-R) (Lord et al., 1994) and Autism Diagnostic Observation Schedule (ADOS) (Lord et al., 1989). Each proband also completed the SSC protocol, which included clinical, medical, behavioral, and family histories; physical, neurologic, and dysmorphology examinations (conducted for a subset of the probands). The 51 phenotypes examined in this work span ASD specific core-symptom measures, cognitive and adaptive functioning, behavioral problems, neurological indicators, and dysmorphic biomarker. The dysmorphic biomarker phenotype, quantified using the Autism Dysmorphology Measure (Miles et al., 2008), distinguishes complex autism (dysmorphic and/or microcephalic) (Miles, 2015) from essential autism (non-dysmorphic and not microcephalic). Given that the distinction between complex and essential autism is important in dissecting the ASD etiologic heterogeneity (Miles, 2015; Spencer et al., 2018), we restricted the sample analyzed in this work to only the 560 probands who underwent dysmorphology examinations (Zhao et al., 2019; Matta et al., 2021). All the 51 phenotype markers utilized in this work are summarized in Table 1. The phenotype markers are scored using widely varying scales, and were normalized using the min-max method prior to graph construction.

**Table 1:** Description of 51 ASD phenotype features.

**Cognitive and adaptive functions**	**Language and communication**
Vineland socialization	Word delay
Vineland daily living	Phrase delay
Vineland overall score	Regression
Nonverbal IQ	Vineland communication
Overall IQ	ADI-R overall language
	
**Facial biomarker**	
Dysmorphic (Yes/No)	
**Behavioral problems**	
**Repetitive behavior scale (RBS) scores**	**Abberant behavior checklist scores**
RBS self injurious	ABC inappropriate speech
RBS stereotyped behavior	ABC lethargy
RBS compulsive behavior	ABC irritability
RBS sameness behavior	ABC stereotype
RBS restricted behavior	ABC hyperactivity
RBS ritualistic behavior	ABC total score
RBS overall score	
**Social responsiveness scale (SRS) parent scores**	**Social responsiveness scale teacher scores**
SRS-P awareness	SRS-T awareness
SRS-P cognition	SRS-T cognition
SRS-P communication	SRS-T communication
SRS-P mannerisms	SRS-T mannerisms
SRS-P motivation	SRS-T motivation
SRS-P overall T score	SRS-T overall T Score
	
CBCL^*a*^ externalizing T score	CBCL^*a*^ internalizing T score
	
**Genetic indicators**	
BAPQ^*b*^ overall average (mother)	BAPQ^*b*^ overall average (father)
	
**ASD-specific symptom scores**	
**Autism diagnostic observation schedule scores**	**Autism diagnostic interview-revised scores**
ADOS communication	ADI-R nonverbal communication
ADOS communication and social	ADI-R socialization
ADOS reciprocal social	ADI-R abnormality evidence
ADOS social affect ADOS restricted and repetitive behavior	ADI-R restricted and repetitive behavior
ADOS calibrated severity score (CSS)	
ADOS module	

^*a*^CBCL, child behavior checklist. ^*b*^BAPQ, broad autism phenotype questionnaire.

The SSC genotype data are derived from DNA specimens that were genotyped using the Illumina mv1, mv3, and omni2.5 SNP genotyping chip arrays. Similar to Zhao et al. (2019), we applied a data-driven approach to extract a subset of the available SNPs based on the strength of known association with the disease studied, ASD in this context. We utilize information from the genome-wide SNP prioritization analysis conducted in Spencer et al. (2018). Their analysis ranked the entire set of SNPs available by strength of association as ordered by increasing values of *p*-value (the smaller the value, the stronger the association). Using this ranking, we selected the top most significant SNPs based on a *p*-value threshold. In addition, SNPs that had the same allele representation across all probands were filtered out, as these SNPs contain no discriminant information. This yielded a set of 14,564 SNPs, based on a *p*-value threshold of < 0.1, utilized for subsequent analysis.

### 2.2. Graph construction

Let *D*_P_ and *D*_SNP_ denote the phenotype and SNP genotype data, respectively. Our goal is to construct a phenotype-phenotype (p-p) graph in which the nodes represent the phenotypes and the edges quantify a degree of relationship between adjacent phenotypes. The most explicit way to generate a p-p graph is to construct the graph directly from a matrix of phenotype features over the entire proband sample. The phenotype matrix obtained from *D*_P_ is of size *m* × *p* where *m* denotes the number of probands (560) and *p*, the number of phenotypes (51). We refer to the resulting p-p graph as a *direct* graph, *G*_*D*_, since it is generated directly from proband-phenotype data profiles. Any missing phenotype data for a given proband is imputed using the mean value. The distance between each pair of phenotype vectors in the matrix is calculated using two functions, Euclidean and cosine. Based on these distances, k-nearest neighbors (kNN) graphs are created, with each phenotype node being connected to its *k* nearest neighbors. The nearest-neighbor relationship is not necessarily symmetric, implying directed graphs. The graphs are then converted to undirected graphs. The resulting graphs have 51 nodes, where each node corresponds to a phenotype, and each node is connected to at least *k* neighbors. Separate graphs are constructed for *k* = 3 to 9. Thus, in the *G*_*D*_ graphs, there exists an edge between two phenotypes if a subset of the probands share similar phenotype traits where the degree of similarity shared is influenced by *k*. Note that in this study none of the samples are genetically related, so that this possibility is not considered in graph construction. Our graph model also does not account for other types of confounding factors, such as sex, age, geographical location, or socioeconomic status.

Inspired by the PheWAS approach, we also construct another p-p graph utilizing the SNP data. We refer to this graph as the *indirect* graph, *G*_*I*_, since it is generated from the outcome of the PheWAS analysis applied to SNP-phenotype data. Specifically, a multi-trait mixed regression model (LIMIX; Lippert et al., 2014) is applied to both SNP and phenotype matrices to emulate the PheWAS model. It combines the two matrices to yield a measure of the correlation between phenotypes and SNPs. The *G*_*I*_ p-p graph is then obtained using the shared SNP associations identified by the PheWAS-inspired analysis. Here the LIMIX regression model would compensate for genetic relatedness, although none is present in our sample. Phenotypic confounding factors are unknown and not part of the data, and our model does not account for them. To construct the *indirect* graph, *D*_SNP_ is encoded into a high dimensional SNP matrix, in which the entries identify the nature of the variant quantifying the proband's risk allele to the reference allele. The resulting SNP matrix is of size *m* × *n* where *n* denotes the number of SNPs (14,564), while the phenotype matrix (same as utilized for *G*_*D*_ graph) is of size *m* × *p*. This yields a matrix of scores quantifying the association between phenotypes and SNPs. In addition, we extract a matrix of *p*-values measuring the probability of phenotype-SNP correlation, where a smaller *p*-value indicates a higher probability of correlation. Unlike in previous work (Matta et al., 2021), the matrix of *p*-values is not filtered based on any threshold. In contrast to Matta et al. (2021), where the filtered matrix was fed directly into the clustering model, we convert this matrix to a kNN graph. The kNN graphs are derived from the *p*-values matrix using two distance functions, Euclidean and cosine (same as in the *G*_*D*_ graph). The resulting graphs have 51 nodes, where each node corresponds to a phenotype, and each node is connected to at least *k* nodes for *k* = 3 to 9. In the *G*_*I*_ graph, two phenotype nodes share an edge if they are likely to have shared similar underlying SNP associations.

### 2.3. Construction of fused graphs

In this work, we are interested in a novel methodology for incorporating the genotype information in the construction of the p-p network. The strategy is to combine the two p-p graphs (*direct* and *indirect*) in a weighted manner so we can evaluate the impact. The indirect (*G*_*I*_) and direct (*G*_*D*_) graphs are combined as follows. First, the graphs are converted to their corresponding adjacency matrices (*A*_*I*_ and *A*_*D*_), where an entry of 1 in the matrix indicates an edge, and 0 indicates no edge. A weighted matrix, *W*, is then generated using the formula


(1) $$\begin{array}{l} W=\alpha \times A_{D}+\left(1-\alpha \right)\times A_{I} \end{array}$$

where α is a weighting parameter which varies between 0.0 and 1.0. This enables construction of a mixed, weighted graph, in which *A*_*I*_ and *A*_*D*_ can be combined in different proportions. *W* is equivalent to *A*_*I*_ when α = 0, and likewise, to *A*_*D*_ when α = 1. Both adjacency matrices contribute equally to *W* when α = 0.5. *W* is considered as the adjacency matrix for the constructed weighted, undirected, fused graphs *G*_α_.

### 2.4. Cluster analysis and evaluation

We perform clustering on the fused graphs to identify meaningful phenotype-phenotype associations, particularly those of clinical importance based on shared underlying genetic etiology. Clustering the graphs should yield subgroups of phenotypes that are most similarly linked in conjunction with the SNP information (applied in varying degrees). Three different graph-based clustering methods are utilized: Louvain clustering (Blondel et al., 2008), the Leiden algorithm (Traag et al., 2018), and Node-Based Resilience Clustering (NBR-Clust) (Matta et al., 2018a) with integrity (Barefoot et al., 1987). Louvain clustering (Blondel et al., 2008) is a well-known algorithm based on optimizing modularity. It has low time complexity and is frequently employed for clustering large datasets. The Leiden algorithm (Traag et al., 2018) represents an improvement over Louvain as it converges to a partition in which all subsets of all communities are locally optimally assigned. It is faster than Louvain, uncovers better partitions, and yields communities that are guaranteed to be connected. The NBR-Clust algorithm (Matta et al., 2018a) uses network resilience measures to partition a given graph into clusters by identifying an attack set of nodes *S*∈*V* whose removal partitions the network into some number of disconnected components. In this work, the resilience measure is integrity, which is approximated using betweenness centrality (Matta, 2017). For all these clustering methods, the number of clusters is not specified *a priori*.

Clustering is a multi-optimization problem which implies that more than one solution could exist. It is imperative to have an objective means to evaluate the results and determine the most optimal solution. We apply six varied graph-based metrics (Yang and Leskovec, 2015) to objectively quantify the connectivity characteristics of the given graph based on the edges and nodes. Given that a cluster in an undirected graph *G*(*V, E*) is a set of nodes *S*, each metric can be defined as a function *f*(*S*) that characterizes how cluster-like the connectivity of the nodes in *S* are. Let *n*_*S*_ be the number of nodes in *S* and *m*_*S*_, the number of edges in *S*; *m*_*S*_ = |(*u, v*)∈*E*:*u*∈*S, v*∈*S*|. Let *c*_*S*_ be the number of edges on the boundary of *S*, *c*_*S*_ = |(*u, v*)∈*E*:*u*∈*S, v*∉*S*|. Newman-Girvan modularity, given by $f\left(S\right)=\frac{1}{m}\underset{c\in S}{\sum }\left(m_{S}-\frac{{\left(2m_{S}+c_{S}\right)}^{2}}{4m}\right)$, is the difference between the fraction of edges within a cluster and the expected number of such edges if distributed according to a null model. Erdos-Renyi modularity [$f\left(S\right)=\frac{1}{m}\underset{c\in S}{\sum }\left(m_{S}-\frac{m\left(n_{S}\right)\left(n_{S}-1\right)}{n\left(n-1\right)}\right)$] is a variation of Newman-Girvan based on the assumption that the vertices in a network are randomly connected with a constant probability *p*. Expansion is the proportion of edges per node pointing outside the cluster, given by $f\left(S\right)=\frac{c_{S}}{n_{S}}$. Cut-ratio is the fraction of edges, out of all possible edges, that leave the cluster, given by $f\left(S\right)=\frac{c_{S}}{n_{S}\left(n-n_{S}\right)}$. Conductance measures the fraction of total edge volume pointing outside the cluster, i.e., $f\left(S\right)=\frac{c_{S}}{2m_{S}+c_{S}}$. Average out degree fraction is the average fraction of edges of the nodes in a cluster that point outside the cluster itself, i.e., $f\left(S\right)=\frac{1}{n_{S}}\underset{u\in S}{\sum }\frac{\left|\left\{\left(u,v\right)\in E:v\notin S\right\}\right|}{d\left(u\right)}$, where *d*(*u*) is the degree of node *u*.

We utilize an ensemble ranking approach to leverage these varied measures to determine the optimal results. In this ensemble ranking approach (adapted from Nguyen et al., 2018), the score of each measure is computed for each clustering result. Based on these scores, the graphs are ranked, where the best scoring graph is ranked number 1, and graphs with worse scores are given larger numerical rankings. The overall score for each graph is obtained by summing the graph's rank over all six metrics, where a lower value indicates a more optimal result.

### 2.5. Model significance and interpretation

After identifying the optimal clustering results based on varied graph properties, the final step is to evaluate the significance of the results by analyzing the biological relevance of each cluster of phenotypes and the associated sets of SNPs. Our overall goal is to identify the set of discriminant genes for its corresponding subgroup of phenotypes from the clustered p-p graphs. To understand the shared properties of their phenotype members and the relevance of the important genes represented, each cluster of phenotypes is characterized using the PheWAS analysis results, similar to Matta et al. (2021). If a cluster contains more than three phenotypes with the same significant SNP (based on the *p*-value), that SNP is considered relevant to the cluster. The exact number of occurrences of the SNP is noted as well. For these SNPs, their respective gene information is obtained and analyzed to see if they are known to have significant associations with the phenotypes in question or ASD in general.

To determine the relevance of the gene with ASD, our preliminary work (Matta et al., 2021) had focused strictly on an extensive literature review to quantify the strength of the association, which had some flaws. In this current work, we employ a more rigorous approach by incorporating the gene scoring process from the SFARI gene database (Abrahams et al., 2013) along with the literature review. This is a more stringent process as the gene scoring system takes into account all available evidence supporting a gene's relevance to ASD risk and places each gene into a category reflecting the overall strength of that evidence. ASD genes could be classified as syndromic (S), category 1 (high confidence), category 2 (strong candidate), or category 3 (suggestive evidence).

## 3. Results

We are interested in evaluating the effect of the fused graphs on the clustering results and clinical relevance. The weighting parameter for the fused graph, α, is varied from 0 to 1 (0:0.1:1). Each kNN (*k* = [3:1:9]) graph is constructed using 2 distance metrics (Euclidean and cosine) and three clustering methods are applied. From the preliminary evaluation of the clustering results using the graph based metrics, we observed that NBR-Clust did not produce any top results, while Louvain and Leiden produced highly similar outcomes. As Leiden is known to be an improvement on Louvain, which mitigates the well-known resolution effect, we chose to focus on the Leiden clustering results for the remainder evaluation and analysis.

**Figure 2:**
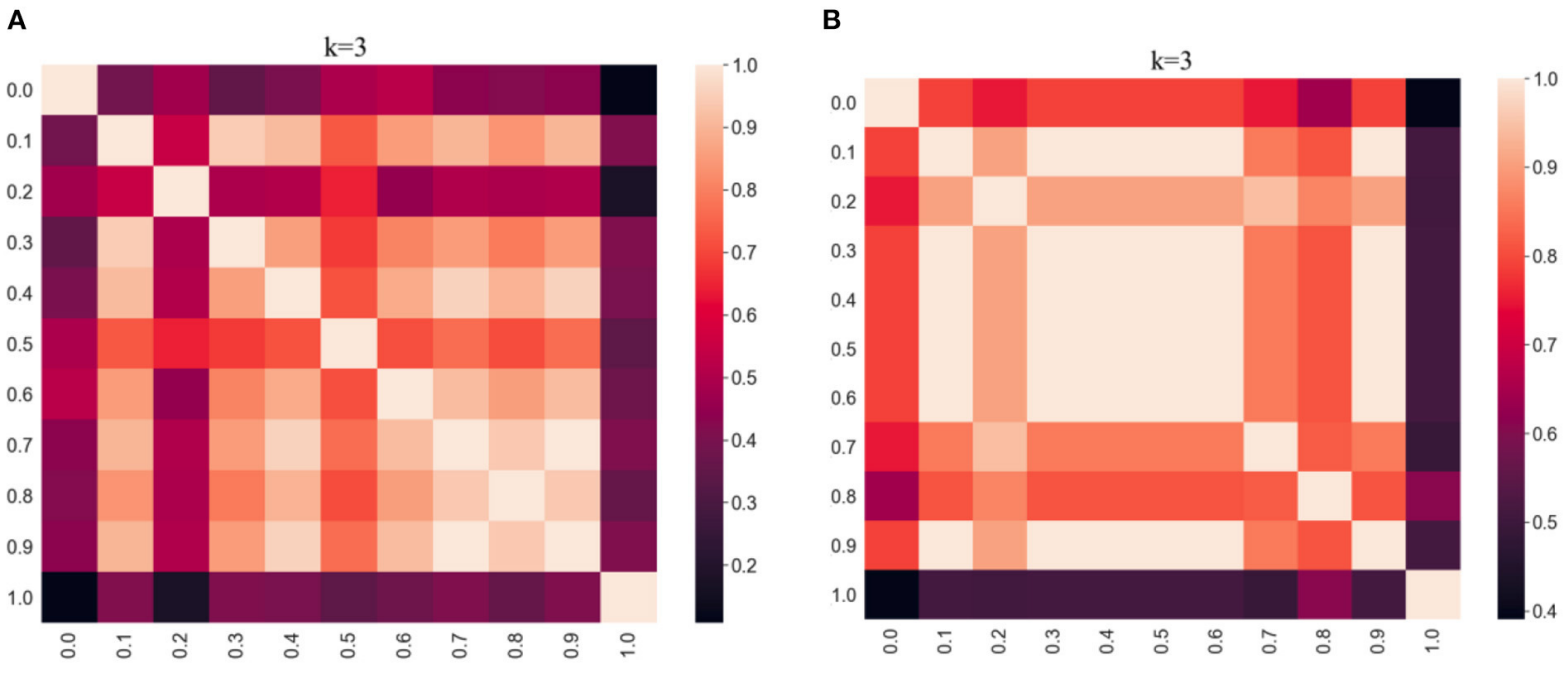
Heatmap evaluation of similarity between clustering results (k = 3, kNN graphs, α = [0:0.1:1]) per distance metric (Euclidean vs. cosine) using adjusted Rand index. **(A)** Results using Euclidean. **(B)** Results using Cosine.

The clustering results obtained by varying α values between 0 and 1 were compared using the adjusted Rand index (ARI), which is a simple way of determining the similarity of two clusterings. This allows us to evaluate the effect of varying the weighting parameter on the clustering results. A high ARI value indicates that the compared partitions have a high degree of similarity. The ARI results are visualized using heatmaps in Figure 2 per distance metric. In the heatmap, the lighter the color, the higher the degree of similarity between the results. The commutative property of the ARI function assures a score of 1.0 along the diagonal of both heatmaps, and both are symmetric. From Figure 2, we observe that the darkest regions are along the edges of the heatmaps for both distances which implies that the results are very different for α = 0.0 compared to α = 1.0. However, it appears that the effect of α is less pronounced with the cosine distance metric as there are relatively more dark regions in Figure 2A (Euclidean) compared to Figure 2B (cosine).

To determine the set of optimal clustering results from the fused (*G*_α_), indirect (*G*_*I*_), and direct (*G*_*D*_) p-p graphs (for both Euclidean and cosine distances), we applied six graph-based evaluation metrics using the ensemble ranking approach (see Section 2.4). Our goal is to compare results obtained from the fused graphs to those from the direct and indirect graphs per type of distance measure employed. Therefore, the clustering results were ranked separately in three categories: α = 0.0, α = 1.0, and α = [0.1:0.1:0.9]. The kNN graphs with *k* varied from 3 to 9, yielded a set of seven graphs for the direct and indirect graphs, respectively, per each distance measure while the combinations of *k* and α values produced 63 fused graphs per distance measure. The top results per category are shown in Table 2. For the fused graph, we present the top two for each distance measure, since we had many more graphs to rank.

**Table 2:** Top ranking graphs based on weighted combination of metrics.

**Graph (k/α/metric)**	**Overall rank**	**Erdos-Renyi modularity**	**Newman-Girvan modularity**	Average ODF^*^	**Conductance**	**Cut ratio**	**Expansion**
**Fused (*G*_α_): α=[0.1:0.1:0.9]**						
3/0.3/Euclidean	9	0.476 (2)	0.493 (2)	2.448 (1)	0.326 (2)	0.060 (1)	2.448 (1)
3/0.5/Euclidean	14	0.480 (1)	0.494 (1)	2.474 (3)	0.328 (4)	0.061 (2)	2.474 (3)
3/0.2/Cosine	10	0.560 (1)	0.598 (3)	1.649 (1)	0.276 (3)	0.039 (1)	1.649 (1)
3/0.6/Cosine	11	0.560 (1)	0.592 (4)	1.649 (1)	0.276 (3)	0.039 (1)	1.649 (1)
**Indirect (*G*_*I*_): α = 0**						
3/0.0/Euclidean	6	0.691 (1)	0.670 (1)	0.465 (1)	0.110 (1)	0.011 (1)	0.465 (1)
3/0.0/Cosine	7	0.694 (1)	0.677 (1)	0.596 (1)	0.133 (2)	0.014 (1)	0.596 (1)
**Direct (*G*_*D*_): α = 1**						
3/1.0/Euclidean	6	0.732 (1)	0.708 (1)	0.470 (1)	0.109 (1)	0.011 (1)	0.470 (1)
4/1.0/Cosine	8	0.610 (2)	0.573 (2)	0.983 (1)	0.165 (1)	0.024 (1)	0.983 (1)

^*^ODF, Out degree fraction. Individual ranks are indicated in parenthesis.

The best possible overall score under our ranking system is 6. As can be observed from Table 2, the direct and indirect graphs seem to yield better overall rank scores compared to the fused graphs. However, we need to keep in mind that they were ranked separately and had a much smaller pool. The rank score may not translate directly in comparing one pool to another but is useful for ranking results within a given pool. The top two results for the cosine fused graphs are very closely ranked (10 vs. 11). The individual metrics all yielded the same value except for Newman-Girvan modularity. Both graphs (*k* = 3 α = 0.2 and α = 0.6) also produced identical clustering results. The slight difference in the modularity score was a result of minimal differences in the edges. It is interesting to note that most of the optimal results (except one) were obtained with *k*=3. This aligns with previous evidence presented in favor of the use of a minimal connectivity parameter k in the construction of kNN graphs (Matta et al., 2018b).

We compared the clustering results for the highest ranked graph per subcategory in Table 3 across all 51 phenotype features (see Table 1) that span ASD-specific symptoms, cognitive and adaptive functions, language and communication, behavioral problems, genetic indicators, and facial biomarker. The similar clusters across results are matched by color, for ease of comparison. Interestingly, in each category, there were both 5 and 6 clusters results. The cosine metric yielded 6 cluster results for both the indirect and fused graphs while the Euclidean produced a 6 cluster result for the direct graph. Based on the visual inspection of the clusters, we observe that for both the indirect and fused graphs, the 6 cluster results appeared most aligned with the known subgroups of the phenotype features. It seems that the cosine distance resulted in more clearly defined clusters. For the remainder of this paper, for brevity, we conduct the model interpretation analysis for the cosine fused graph (*G*_α = 0.2_), cosine indirect graph (*G*_*I*_), and Euclidean direct graph (*G*_*D*_) clustering results. We also compare the outcomes to our prior work (Matta et al., 2021).

**Table 3:** Phenotype clusters identified by color for the top ranking cosine and Euclidean graphs in the indirect, fused, and direct categories.

	**Indirect (** ***G***_*****I*****_ **)**		**Fused (** ***G***_**α**_ **)**		**Direct (** ***G***_*****D*****_ **)**	
**Features**	**Cosine k = 3 α = 0.0**	**Euclidean k = 3 α=0.0**	**Cosine k = 3 α = 0.2**	**Euclidean k = 3 α = 0.3**	**Cosine k = 4 α = 1.0**	**Euclidean k = 3 α = 1.0**
	**6 clusters**	**5 clusters**	**6 clusters**	**5 clusters**	**5 clusters**	**6 clusters**
**ASD-specific symptom scores**ADOS communication							C6	C5	C4	C4	C3	C1
ADOS communication and social	C6	C5	C4	C4	C3	C4
ADOS reciprocal social	C6	C5	C4	C4	C3	C5
ADOS social affect	C6	C5	C4	C4	C3	C4
ADOS restricted and repetitive behavior	C6	C5	C4	C1	C3	C1
ADOS calibrated severity score (CSS)	C6	C5	C4	C4	C3	C5
ADOS module	C1	C1	C1	C1	C4	C1
ADI-R nonverbal communication	C1	C1	C1	C4	C2	C5
ADI-R socialization	C1	C1	C1	C2	C2	C3
ADI-R abnormality evidence	C1	C1	C2	C1	C2	C1
ADI-R restricted and repetitive behavior	C2	C2	C2	C4	C2	C5
**Cognitive and adaptive functions**Vineland socialization							C1	C1	C1	C2	C4	C2
Vineland daily living	C1	C1	C1	C2	C4	C2
Vineland overall score	C1	C1	C1	C2	C4	C2
Nonverbal IQ	C1	C2	C1	C2	C4	C2
Overall IQ	C1	C1	C1	C2	C4	C2
**Language and communication**ADI-Revised overall language							C1	C1	C1	C2	C2	C6
Word delay	C1	C1	C1	C2	C2	C6
Phrase delay	C1	C1	C1	C2	C2	C6
Regression	C1	C1	C4	C2	C3	C6
Vineland communication	C1	C1	C1	C2	C4	C2
**Behavioral problems**ABC^*a*^ inappropriate speech							C2	C2	C5	C1	C1	C1
ABC lethargy	C2	C3	C2	C3	C1	C5
ABC irritability	C3	C2	C5	C3	C1	C4
ABC stereotype	C3	C2	C3	C1	C1	C1
ABC hyperactivity	C3	C2	C5	C3	C1	C3
	**6 clusters**	**5 clusters**	**6 clusters**	**5 clusters**	**5 clusters**	**6 clusters**
ABC total score	C3	C2	C5	C3	C1	C2
CBCL^*b*^ externalizing T score	C3	C2	C5	C3	C2	C2
CBCL internalizing T score	C2	C3	C2	C3	C2	C2
RBS^*c*^ self injurious	C3	C2	C5	C1	C1	C1
RBS stereotyped behavior	C4	C2	C3	C1	C1	C1
RBS compulsive behavior	C4	C2	C3	C1	C1	C1
RBS sameness behavior	C4	C2	C3	C1	C1	C5
RBS restricted behavior	C4	C2	C3	C1	C1	C1
RBS ritualistic behavior	C4	C2	C3	C1	C1	C1
RBS overall score	C4	C2	C3	C1	C1	C3
SRS^*d*^-P awareness	C2	C3	C2	C3	C1	C4
SRS-P cognition	C2	C3	C2	C3	C1	C3
SRS-P communication	C2	C3	C2	C3	C1	C3
SRS-P mannerisms	C2	C3	C3	C3	C1	C3
SRS-P motivation	C2	C3	C2	C3	C1	C3
SRS-P overall T score	C2	C3	C2	C3	C2	C2
SRS-T awareness	C5	C4	C6	C5	C5	C4
SRS-T cognition	C5	C4	C6	C5	C5	C4
SRS-T communication	C5	C4	C6	C5	C5	C3
SRS-T mannerisms	C5	C4	C6	C5	C5	C4
SRS-T motivation	C5	C4	C6	C5	C5	C4
SRS-T overall T score	C5	C4	C6	C5	C2	C2
**Genetic indicators**						
BAPQ^*e*^ mother	C2	C4	C2	C1	C2	C1
BAPQ father	C2	C1	C2	C1	C2	C1
**Facial biomarker**Dysmorphic (Yes/No)							C2	C4	C2	C2	C2	C6

^*a*^ABC, aberrant behavior checklist. ^*b*^CBCL, child behavior checklist. ^*c*^RBS, repetitive behavior scale. ^*d*^SRS, social responsiveness scale. ^*e*^BAPQ, broad autism phenotype questionnaire. Colors denote similar clusters aligned across results.

## 4. Biological relevance of results

Given that we are interested in evaluating the effect of clustering the phenotypes using the fused approach compared to simply direct or indirect approach in identifying relevant genotype-phenotype associations, the final step involves evaluating the significance of the results. This involves analyzing the biological relevance of each cluster of phenotypes and their associated sets of SNPs/discriminant genes (see Section 2.5). The summary of the analysis for each of the three graphs is shown in Tables 4–6. In each of the tables, the list of relevant SNPs along with the exact number of occurrences and their associated genes is presented for each cluster of phenotypes. Genes identified by the SFARI gene database as having potential relevance to ASD risk are shown in bold.

**Table 4:** Important genes per phenotype cluster for cosine *k* = 3 α = 0.2 fused graph (*G*_α = 0.2_).

**Cluster number**	**Phenotypes**	**SNPs (occurrences)**	**Important genes**
1	ADOS module, ADI-R scores (nonverbal communication, socialization, revised overall language), Vineland scores (socialization, daily living, overall, communication), nonverbal IQ, overall IQ, word delay, phrase delay, BAPQ father	rs1864086 (9), rs7174994 (8), rs11665578 (8), rs6765578 (8), rs948396 (8), rs7192876 (8), rs9826422 (7), rs12715538 (7), rs17042412 (7), rs1563119 (7), rs9637712 (7), rs1792136 (7), rs11215264 (7), rs7984277 (6), rs10173578 (6), rs700873 (6), rs1718031 (6), rs1522026 (6), rs855017 (6)	RTTN, RARB, TENM4, ZDHHC7, LOC105369506, MGAT4A
2	ADI-R scores (abnormality evidence, restricted and repetitive behavior), ABC lethargy, CBCL internalizing T score, SRS-P scores (awareness, cognition, mannerisms, motivation, overall T score), BAPQ scores (mother, father), dysmorphic	rs17141324 (6), rs4653939 (4), rs2087121 (4), rs4653942 (4), rs16930719 (4), rs17328164 (4), rs16085 (4), rs826021 (4), rs855025 (4), rs700874 (4), rs7034595 (4), rs7863199 (4), rs2109217 (4), rs35765056 (4), rs13013415 (3), rs12005201 (3)	OBSCN, CCDC77, RNF38, WDR33
3	ABC stereotype, RBS scores (stereotyped behavior, compulsive behavior, sameness behavior, restricted behavior, ritualistic behavior, overall score), SRS-P mannerisms	rs9945776 (6), rs6943423 (5), rs9582807 (5), rs6707140 (5), rs9959803 (5), rs4653939 (4), rs2087121 (4), rs4653942 (4), rs6555976 (4), rs2024672 (4), rs17329797 (4), rs593949 (4), rs2461697 (4), rs6582086 (4), rs2220158 (4)	DCC, OBSCN, TSHZ2
4	ADOS scores (communication, communication and social, reciprocal social, social affect, restricted and repetitive behavior, calibrated severity score), regression	rs16860907 (5), rs1933099 (5), rs3753938 (5), rs12476865 (5), rs2535370 (5), rs1927636 (5), rs16973788 (5), rs8084578 (5), rs4851946 (4), rs6534010 (4), rs10006459 (4), rs7688689 (4), rs12505042 (4), rs1599167 (4), rs2290847 (4)	XCL1, SEMA3E, LRBA
5	ABC scores (inappropriate speech, irritability, hyperactivity, total score), CBCL exernalizing T score, RBS self injurious	rs3828139 (5), rs2195086 (4), rs17552548 (4), rs4113420 (4), rs10789439 (3), rs441196 (3), rs4510173 (3), rs6707140 (3), rs250791 (3), rs11961507 (3), rs17586672 (3), rs10098925 (3), rs13293564 (3), rs12573176 (3), rs7326004 (3)	ST3GAL3, CCDC149, FAM155A, KDM4A, SLC8A1-AS1, DIAPH1, UNC13B
6	SRS-T scores (awareness, cognition, communication, mannerisms, motivation, overall)	rs716897 (6), rs6687487 (5), rs10017022 (5), rs4705000 (5), rs9314498 (5), rs7871600 (5), rs12306561 (5), rs9566309 (5), rs1884606 (5), rs6135739 (5), rs2144885 (5), rs6810871 (4), rs771662 (4), rs655089 (4), rs9576449 (4)	EPHB2, SGCD, CSMD1, LINGO2, KSR2, KIF16B

Genes identified by the SFARI gene database as having potential relevance to ASD risk are shown in bold.

**Table 5:** Important genes per phenotype cluster for cosine *k* = 3 α = 0 indirect graph (*G*_*I*_).

**Cluster number**	**Phenotypes**	**SNPs (occurrences)**	**Important genes**
1	ADOS module, ADI-R scores (nonverbal communication, socialization, abnormality evidence. revised overall language), Vineland scores (socialization, daily living, overall, communication), nonverbal IQ, overall IQ, word delay, phrase delay, regression	rs1864086 (9), rs7174994 (8), rs11665578 (8), rs6765578 (8) rs948396 (8), rs7192876 (8), rs9826422 (7), rs12715538 (7), rs17042412 (7), rs1563119 (7), rs9637712 (7), rs1792136 (7), rs11215264 (7), rs7984277 (6), rs10173578 (6)	RTTN, RARB, TENM4, ZDHHC7, LOC105369506, MGAT4A
2	ADI-R restricted and repetitive behavior, ABC scores (inappropriate speech, lethargy), CBCL internalizing T score, SRS-P scores (awareness, cognition, communication, mannerisms, motivation, overall), BAPQ (mother and father), dysmorphic	rs17141324 (7), rs4653939 (5), rs2087121 (5), rs4653942 (5), rs16085 (5), rs11650915 (5), rs826021 (5) rs700874 (5), rs7034595 (5), rs7863199 (5), rs2109217 (5), rs35765056 (5), rs16930719 (4), rs17328164 (4), rs4890127 (4), rs1960715 (4)	OBSCN, RNF38, CCDC77
3	ABC scores (irritability, stereotype, hyperactivity, total), CBCL externalizing T score, RBS self injurious	rs3828139 (4), rs2195086 (4), rs17552548 (4), rs4113420 (4), rs2279867 (4), rs10789439 (3), rs441196 (3), rs4510173 (3), rs6707140 (3), rs250791 (3), rs11961507 (3), rs17586672 (3), rs10098925 (3), rs13293564 (3)	ST3GAL3, CCDC149, FAM155A, EMP2, KDM4A, SLC8A1, DIAPH1, UNC13B
4	RBS scores (stereotyped behavior, compulsive behavior, sameness behavior, restricted behavior, ritualistic behavior, overall)	rs9945776 (6), rs9582807 (5), rs9959803 (5), rs6943423 (4), rs6707140 (4), rs593949 (4), rs2461697 (4), rs6582086 (4), rs2220158 (4), rs17329797 (3), rs1471655 (3), rs16885002 (3), rs7732186 (3), rs16885032 (3), rs6555976 (3)	DCC
5	SRS-T scores (awareness, cognition, communication, mannerisms, motivation, overall)	rs716897 (6), rs6687487 (5), rs10017022 (5), rs4705000 (5), rs9314498 (5), rs7871600 (5), rs12306561 (5), rs9566309 (5), rs1884606 (5), rs6135739 (5), rs2144885 (5)	EPHB2, SGCD, CSMD1, LINGO2, KSR2, KIF16B
6	ADOS scores (communication, communication and social, reciprocal social, social affect, restricted and repetitive behavior, calibrated severity score)	rs16860907 (5), rs1933099 (5), rs3753938 (5), rs12476865 (5), rs2535370 (5), rs1927636 (5), rs16973788 (5), rs8084578 (5), rs4851946 (4), rs6534010 (4), rs10006459 (4), rs7688689 (4), rs12505042 (4), rs1599167 (4), rs2290847 (4)	XCL1, SEMA3E, LRBA

Genes identified by the SFARI gene database as having potential relevance to ASD risk are shown in bold.

**Table 6:** Important genes per phenotype cluster for Euclidean *k* = 3 α = 1 direct graph (*G*_*D*_).

**Cluster number**	**Phenotypes**	**SNPs (occurrences)**	**Important genes**
1	ADOS scores (communication, restricted and repetitive behavior, module), ADI-R abnormality evidence, ABC scores (inappropriate speech, stereotype), RBS scores (self injurious, stereotyped behavior, compulsive behavior, restricted behavior, ritualistic behavior), BAPQ scores (mother, father)	rs16973788 (5), rs9325190 (5), rs6555976 (4), rs6943423 (4), rs17757512 (4), rs9582807 (4), rs12465007 (4), rs1471655 (4), rs7335101 (4), rs9945776 (4), rs1581586 (3), rs6703099 (3), rs9284874 (3), rs10520104 (3), rs1864086 (3)	CFAP65, SPATA13, DCC, SH3D19
2	Vineland scores (socialization, daily living, overall, communication), nonverbal IQ, overall IQ, ABC total score, CBCL scores (externalizing T score, internalizing T score), SRS-P overall T score, SRS-T overall T score	rs4653939 (8), rs2087121 (8), rs4653942 (8), rs12488867 (6), rs1864086 (6), rs948396 (6), rs6707140 (5), rs855025 (5), rs700874 (5), rs9576452 (5), rs9566309 (5), rs7982105 (5), rs12715538 (5), rs1563119 (5), rs6765578 (5), rs11215264 (5), rs7174994 (5)	LOC105369506, OBSCN, GPR149, TENM4, RARB
3	ADI-R socialization, ABC hyperactivity, RBS overall score, SRS-P scores (cognition, communication, mannerisms, motivation), SRS-T communication	rs7335101 (4), rs826021 (4), rs4653939 (4), rs2087121 (4), rs4653942 (4), rs17141324 (4), rs716897 (3), rs11961507 (3), rs7326004 (3), rs2788856 (3), rs17329797 (3), rs700874 (3), rs795892 (3), rs7575760 (3), rs12005201 (3), rs7034595 (3)	SPATA13, OBSCN, FAM155A, RBPJ, RNF38
4	ADOS scores (communication and social, social affect), ABC irritability, SRS-P awareness, SRS-T scores (awareness, cognition, motivation, motivation)	rs4705000 (4), rs716897 (4), rs519866 (3), rs655089 (3), rs6687487 (3), rs10017022 (3), rs9314498 (3), rs7871600 (3), rs12306561 (3), rs9566309 (3), rs1884606 (3), rs6135739 (3), rs2144885 (3)	SGCD, EPHB2, CSMD1, LINGO2, KSR2, KIF16B
5	ADOS scores (reciprocal social, calibrated severity score), ADI-R scores (nonverbal communication, restricted and repetitive behavior), ABC lethargy, RBS sameness behavior	rs2296698 (3), rs16860907 (3), rs1933099 (3), rs3753938 (3), rs7155706 (3)	ADGRL2, XCL1, SLC35F4
6	ADI-R overall language, word delay, phrase delay, regression, dysmorphic	rs4305581 (3), rs4871046 (3)	None

Genes identified by the SFARI gene database as having potential relevance to ASD risk are shown in bold.

For the fused graph (*G*_α = 0.2_), shown in Table 4, cluster C1 consists of all the features associated with cognitive and adaptive functions, most of the language and communication features (except regression), ADOS module, ADI-R nonverbal communication, and ADI-R socialization. Important genes aligned with this set of phenotypes are RARB, TENM4, ZDHHC7, LOC105369506, and MGAT4A. RARB is a known neurodevelopmental delay gene Doan et al. (2019). ZDHHC7 is deemed to be directly related to possible ASD candidate genes by biological function (Hedges et al., 2012). There is some evidence for association of LOC105369506 (Ruisch et al., 2019) to childhood antisocial behavior, while MGAT4A (Sanders et al., 2017) shows links to schizophrenia. TENM4 is known to play an important role in central nervous system development and could be a candidate gene for schizophrenia (Xue et al., 2019). We observe from Table 3 that *G*_*I*_ C1 overlaps strongly with this cluster, with two additional phenotypes: ADI-R abnormality evidence and regression. Interestingly, these two clusters also have the same important genes (Table 5). Given that the fused graph has an α = 0.2, closer to 0, it is intuitive that the fused graph clusters are relatively closer to the indirect than the direct graph. When compared to the clusters from *G*_*D*_ (Table 3), we observe that *G*_α = 0.2_ C1 overlaps across both the C2 and C6 clusters from *G*_*D*_. From Table 6, C2 and C6 consist of all the cognitive and adaptive function phenotypes, the language and communication phenotypes, as well as ABC total score, CBCL externalizing and internalizing scores, and SRS parent and teacher overall scores. Out of the five genes associated with *G*_*D*_ C2, three of them were also identified in the *G*_α = 0.2_ C1 gene list. One of them, OBSCN, is listed among the genes associated with both *G*_α = 0.2_ clusters C2 and C3. OBSCN is possibly an ASD risk gene (Krupp et al., 2017). It seems to be connected with the SRS parent and teacher overall scores phenotypes.

Cluster C2 of *G*_α = 0.2_ seems more closely associated with phenotypes related to behavioral problems. It consists of ADI-R abnormality evidence, ADI-R restricted and repetitive behavior, ABC lethargy, CBCL internalizing score, all the SRS parent scores except mannerisms, BAPQ father and mother, and dysmorphic facial biomarker. There are several important genes, of which OBSCN has already been mentioned. RNF38 has been identified by the SFARI gene database (Abrahams et al., 2013) with a score of 3, indicating suggestive evidence of a link with ASD. WDR33 is also mentioned in connection with ASD in Stessman et al. (2017) with an exclusive male bias. Interestingly, it is the only gene not also replicated in any cluster of either the direct or indirect graphs. The *G*_α = 0.2_ cluster C2 also overlaps with cluster C2 (*G*_*I*_) and C3 (*G*_*D*_). The *G*_*I*_ cluster C2 does not contain ADI-R abnormality evidence and also includes ABC inappropriate speech and SRS-P mannerisms. All its important genes are a subset of *G*_α = 0.2_ C2 genes (Table 5). The *G*_*D*_ cluster C3 contains the SRS-P scores, except for awareness and overall T score. In addition, it also consists of ADI-R socialization, ABC hyperactivity, RBS overall score, and SRS-T communication. Important additional genes linked with this cluster include SPATA13, FAM155A and RBPJ. SPATA13 has been identified as possibly associated with ASD in males (Kong et al., 2012). Interestingly, these genes also seem to be genetically associated with related disorders such as social behavior (SPATA13, Bourbia et al., 2019), ADHD (FAM155A, Yang et al., 2018; RBPJ, Martin et al., 2014), and impulsive behavior (FAM155A, Vevera et al., 2019).

Cluster C3 of *G*_α = 0.2_ also seems to consist mainly of RBS behavioral problem phenotypes (except for self injurious), as well as ABC stereotype behavior and SRS-P mannerisms. It is associated with the DCC gene which has a SFARI gene score of 2, i.e., a strong candidate for ASD. This cluster is also associated with OBSCN and TSCHZ2 (a gene differentially expressed in Phelan-McDermid syndrome, which has a high risk of ASD; Breen et al., 2020). For comparison, this cluster is similar to the *G*_*I*_ cluster C4, containing the same RBS behavioral scores, but without the ABC stereotype and SRS-P mannerisms phenotypes. The *G*_*I*_ cluster C4 has only DCC as an important gene. These clusters overlap weakly with the *G*_*D*_ cluster C1, which contains all RBS scores, except sameness behavior and overall score. In addition, *G*_*D*_ cluster C1 contains the BAPQ average father and mother scores, some ADOS scores (communication, restricted and repetitive behavior), ADOS module, ADI-R abnormality evidence, ABC inappropriate speech, and ABC stereotype. Like *G*_α = 0.2_ C3 and *G*_*I*_ C4, *G*_*D*_ C1 is associated with DCC. Other important genes are CFAP65, SPATA13, and SH3D19. CFAP65 is a candidate gene for epilepsy (Poirier et al., 2017), which appears to have some asserted genetic association to autism. SPATA13, as discussed in relation to C2 of the fused graph, is a potential relevant ASD gene.

Cluster C4 of *G*_α = 0.2_ appears to be mainly a set of ADOS diagnosis scores. It is made up of all six ADOS scores, except ADOS module (found in C1), along with the regression phenotype. The three genes (LRBA, SEMA3E, and XCL1) associated with this cluster appear to all have some ASD risk relevance. According to the SFARI gene scoring system (Abrahams et al., 2013), LRBA is a category 3 (suggestive evidence) ASD risk gene. XCL1 lies in the region of copy number variants of high interest identified in children with ASD (Davis et al., 2009). SEMA3E (Lin et al., 2018) was found to be upregulated in a family quartet with ASD. From Table 3, this cluster is also replicated in the *G*_*I*_ clustering results (C6) with the same important genes. However, it doesn't seem to overlap with any particular set of clusters when compared to the *G*_*D*_ results. Interestingly, two of these genes (LRBA, SEMA3E) are not found in any of the important genes listed for the direct graph across all its clusters (Table 6).

The smallest cluster in *G*_α = 0.2_ is C5 with 6 phenotypes, consisting of the ABC aberrant behavior scores (inappropriate speech, irritability, hyperactivity, and total score), CBCL externalizing T score, and RBS self injurious. The important genes associated with this cluster are ST3GAL3, CCDC149, FAM155A, KDM4A, SLC8A1-AS1, DIAPH1, and UNC13B. The ST3GAL3 gene is linked to non-syndromic autosomal recessive intellectual disability (Hu et al., 2011) as well as ADHD (Zhao et al., 2018). FAM155A, also identified with clusters C1 and C3 of the direct graph, is associated with ADHD and impulsive behavior disorders (Yang et al., 2018; Vevera et al., 2019). KDM4A has been shown to increase copy number gains in copy number variants associated with ASD (Cogill et al., 2018). DIAPH1 plays a crucial role in human brain development and is linked to microcephaly (Ercan-Sencicek et al., 2015). It is closely related to DIAPH3, an ASD candidate gene (Vorstman et al., 2011). UNC13B is potentially associated with epilepsy (Wang et al., 2021). This cluster overlaps closely with *G*_*I*_ cluster C3, which does not include ABC inappropriate speech but has ABC stereotype (not present in *G*_α = 0.2_ C5). All the genes associated with *G*_α = 0.2_ C5 are a subset of *G*_*I*_ C3 genes, which also contain EMP2.

Cluster C6 of *G*_α = 0.2_ consists of SRS teacher component and overall scores. It is identical to cluster C5 of *G*_*I*_ (Table 5). It overlaps slightly with cluster C4 of *G*_*D*_ (Table 6), which also includes other phenotypes such as ADOS communication & social, ADOS social affect, SRS-P awareness, and ABC irritability. The genes associated with C6 of *G*_α = 0.2_ are EPHB2, KSR2, CSMD1, SGCD, LINGO2, KIF16B. According to the SFARI gene scoring system (Abrahams et al., 2013), EPHB2, KSR2, and CSMD1 belong to category 3 (suggestive evidence for ASD). CSMD1 is also associated with cognitive function (Athanasiu et al., 2017). Copy number variants of LINGO2 have been found in ASD cases (Williams et al., 2019), and LINGO2 deletions have been reported in individuals with developmental delay, autistic behavior, and craniofacial abnormalities (Jansen et al., 2019). Bi-allelic variants in the KIF16B gene may be linked to autosomal recessive intellectual disability syndrome (Alsahli et al., 2018). We observe from the clustering results of *G*_*D*_ (Table 3), that its cluster C5 does not align with any of the clusters from the fused or indirect graphs. This cluster consists of some ADOS and ADI-R scores, ABC lethargy, and RBS sameness behavior phenotypes. The associated genes are ADGRL2 (a variant of which is linked to severe microcephaly; Vezain et al., 2018), XCL1 and SLC35F4.

The prior work had identified seven (not eight as mentioned) genes with strong previous evidence of association to ASD, as well as 14 genes with weaker previous evidence of links to ASD and other related conditions, based on the literature review presented. Most of these genes are identified in Tables 4–6 other than CACNG4, KRT26, NHEJ1, and TNIK. Upon further investigation of the genes that were deemed to have strong ASD association evidence, we now clarify that genes XCL1, SEMA3E, and SPATA13 are possibly linked to ASD (not strongly associated as previously suggested). The previously stated strong association genes (DIAPH1 and RARB) as well as ST3GAL3, SGCD, and TENM4 have no known verifiable link to ASD but may be associated with related conditions. EPHB2 has suggestive evidence (not strong) for ASD, according to SFARI gene scoring system (Abrahams et al., 2013).

## 5. Conclusion

This paper presented a holistic PheWAS-inspired method to identify meaningful associations between ASD phenotypes and genotypes. We generated p-p graphs utilizing genotype data by fusing direct and indirect graphs based on a weighting parameter α. We compared the outcome to the direct and indirect approaches. The rigorous biological analysis approach taken in this work highlights 28 genes associated with the fused graph clustering results, of which six genes (DCC, RNF38, LRBA, EPHB2, CSMD1, and KSR2) are also identified in the SFARI gene scoring system. The indirect graph also contains these six genes, though the ordering of the phenotype clusters and total genes differ slightly. The direct graph clustering results have five of these genes reflected in the outcomes. Due to the limited ASD simplex sample analyzed, the generalizability of current findings is limited, and we do not present a classification system that fully explains the heterogeneity of ASD. However, our study contains several useful and novel results. First, while ASD is typically diagnosed based on behavioral assessments, this research provides evidence of the utility of including genotype markers in diagnosis. Second, we provide a methodology for identifying meaningful phenotype-phenotype associations, particularly those of clinical importance based on shared underlying genetic etiology. Third, the utility of the methodology of combining genotype and phenotype information is proved empirically by fact that our direct implementation did not discover all the genes that were discovered by incorporating genetic information, including one that the SFARI gene database indicates has suggestive evidence of association with ASD. Fourth, we are able to provide evidence of association that correlates with evidence in the SFARI gene database, strengthening current knowledge. Last, the overall closeness between our results and previously known categorizations helps to both validate existing knowledge and to suggest a path to discovering new genotype-phenotype associations. Future research should include replication with different and larger sample sizes.

## Data availability statement

Qualified researchers can obtain the datasets analyzed for this study upon application to the Simons Foundation Autism Research Initiative. Information about the datasets can be seen at https://www.sfari.org/resource/simons-simplex-collection/, and application can be made at https://base.sfari.org/.

## Ethics statement

The studies involving human participants were reviewed and approved by both the SIUE IRB and the Missouri State IRBs. Written informed consent to participate in this study was provided by the participants' legal guardian/next of kin.

## Author contributions

TO-A, YE-M, and JM designed the study. DD, DY, and SH wrote the software, ran the experiments, and collected the data. JM and TO-A wrote the initial manuscript, with YE-M participating in the final writing. All authors participated in interpretation of the data. All authors contributed to the article and approved the submitted version.

## Funding

Funding for publication fees was provided by the Computer Science Department and the Graduate School at Southern Illinois University Edwardsville.

## Conflict of interest

The authors declare that the research was conducted in the absence of any commercial or financial relationships that could be construed as a potential conflict of interest.

## Publisher's note

All claims expressed in this article are solely those of the authors and do not necessarily represent those of their affiliated organizations, or those of the publisher, the editors and the reviewers. Any product that may be evaluated in this article, or claim that may be made by its manufacturer, is not guaranteed or endorsed by the publisher.

## References

[B1] Abrahams B. S. Arking D. E. Campbell D. B. Mefford H. C. Morrow E. M. Weiss L. A.. (2013). Sfari gene 2.0: a community-driven knowledgebase for the autism spectrum disorders (ASDs). Mol. Autism 4:36. 10.1186/2040-2392-4-36 24090431 PMC3851189

[B2] Alsahli S. Arold S. T. Alfares A. Alhaddad B. Al Balwi M. Kamsteeg E.-J.. (2018). Kif16b is a candidate gene for a novel autosomal-recessive intellectual disability syndrome. Am. J. Med. Genet. Part A 176, 1602–1609. 10.1002/ajmg.a.38723 29736960

[B3] Athanasiu L. Giddaluru S. Fernandes C. Christoforou A. Reinvang I. Lundervold A. J.. (2017). A genetic association study of CSMD1 and CSMD2 with cognitive function. Brain Behav. Immun. 61, 209–216. 10.1016/j.bbi.2016.11.026 27890662

[B4] Barefoot C. Entringer R. Swart H. (1987). Integrity of trees and powers of cycles. Congr. Numer 58, 103–114.

[B5] Binder E. B. (2021). Genotype-phenotype predictions in autism: are we there yet? Am. J. Psychiatry 178, 11–12. 10.1176/appi.ajp.2020.20111589 33384006

[B6] Blondel V. D. Guillaume J.-L. Lambiotte R. Lefebvre E. (2008). Fast unfolding of communities in large networks. J. Stat. Mech. 2008:P10008. 10.1088/1742-5468/2008/10/P1000821517554

[B7] Bourbia N. Chandler P. Codner G. Banks G. Nolan P. M. (2019). The guanine nucleotide exchange factor, SPATA13, influences social behaviour and nocturnal activity. Mammal. Genome 30, 54–62. 10.1007/s00335-019-09800-9 31020388 PMC6491400

[B8] Breen M. S. Browne A. Hoffman G. E. Stathopoulos S. Brennand K. Buxbaum J. D.. (2020). Transcriptional signatures of participant-derived neural progenitor cells and neurons implicate altered Wnt signaling in Phelan-McDermid syndrome and autism. Mol. Autism 11, 1–23. 10.1186/s13229-020-00355-0 32560742 PMC7304190

[B9] Brueggeman L. Koomar T. Michaelson J. J. (2020). Forecasting risk gene discovery in autism with machine learning and genome-scale data. Sci. Rep. 10, 1–11. 10.1038/s41598-020-61288-5 32165711 PMC7067874

[B10] Bruining H. De Sonneville L. Swaab H. De Jonge M. Kas M. van Engeland H.. (2010). Dissecting the clinical heterogeneity of autism spectrum disorders through defined genotypes. PLoS ONE 5:e10887. 10.1371/journal.pone.0010887 20526357 PMC2878316

[B11] Chang J. Gilman S. R. Chiang A. H. Sanders S. J. Vitkup D. (2015). Genotype to phenotype relationships in autism spectrum disorders. Nat. Neurosci. 18, 191–198. 10.1038/nn.3907 25531569 PMC4397214

[B12] Cogill S. B. Srivastava A. K. Yang M. Q. Wang L. (2018). Co-expression of long non-coding RNAs and autism risk genes in the developing human brain. BMC Syst. Biol. 12:91. 10.1186/s12918-018-0639-x 30547845 PMC6293492

[B13] Davis L. Meyer K. Rudd D. Librant A. Epping E. Sheffield V.. (2009). Novel copy number variants in children with autism and additional developmental anomalies. J. Neurodev. Disord. 1, 292–301. 10.1007/s11689-009-9013-z 21547721 PMC3164008

[B14] Denny J. C. Ritchie M. D. Basford M. A. Pulley J. M. Bastarache L. Brown-Gentry K.. (2010). PheWAS: demonstrating the feasibility of a phenome-wide scan to discover gene-disease associations. Bioinformatics 26, 1205–1210. 10.1093/bioinformatics/btq126 20335276 PMC2859132

[B15] Doan R. N. Lim E. T. De Rubeis S. Betancur C. Cutler D. J. Chiocchetti A. G.. (2019). Recessive gene disruptions in autism spectrum disorder. Nat. Genet. 51:1092. 10.1038/s41588-019-0433-8 31209396 PMC6629034

[B16] Ercan-Sencicek A. G. Jambi S. Franjic D. Nishimura S. Li M. El-Fishawy P.. (2015). Homozygous loss of DIAPH1 is a novel cause of microcephaly in humans. Eur. J. Hum. Genet. 23, 165–172. 10.1038/ejhg.2014.82 24781755 PMC4297910

[B17] Fischbach G. D. Lord C. (2010). The simons simplex collection: a resource for identification of autism genetic risk factors. Neuron 68, 192–195. 10.1016/j.neuron.2010.10.006 20955926

[B18] Georgiades S. Szatmari P. Boyle M. Hanna S. Duku E. Zwaigenbaum L.. (2013). Investigating phenotypic heterogeneity in children with autism spectrum disorder: a factor mixture modeling approach. J. Child Psychol. Psychiatry 54, 206–215. 10.1111/j.1469-7610.2012.02588.x 22862778

[B19] Gutiérrez-Sacristán A. Sáez C. De Niz C. Jalali N. DeSain T. N. Kumar R.. (2021). Multi-phewas intersection approach to identify sex differences across comorbidities in 59 140 pediatric patients with autism spectrum disorder. J. Am. Med. Inform. Assoc. 29, 230–238. 10.1093/jamia/ocab144 34405856 PMC8757290

[B20] Hebbring S. J. Schrodi S. J. Ye Z. Zhou Z. Page D. Brilliant M. H. (2013). A pheWAS approach in studying hla-drb1* 1501. Genes Immun. 14, 187–191. 10.1038/gene.2013.2 23392276 PMC3637423

[B21] Hedges D. J. Hamilton-Nelson K. L. Sacharow S. J. Nations L. Beecham G. W. Kozhekbaeva Z. M.. (2012). Evidence of novel fine-scale structural variation at autism spectrum disorder candidate loci. Mol. Autism 3:2. 10.1186/2040-2392-3-2 22472195 PMC3352055

[B22] Hu H. Eggers K. Chen W. Garshasbi M. Motazacker M. M. Wrogemann K.. (2011). ST3GAL3 mutations impair the development of higher cognitive functions. Am. J. Hum. Genet. 89, 407–414. 10.1016/j.ajhg.2011.08.008 21907012 PMC3169827

[B23] Jansen S. van der Werf I. M. Innes A. M. Afenjar A. Agrawal P. B. Anderson I. J.. (2019). De novo variants in FBXO11 cause a syndromic form of intellectual disability with behavioral problems and dysmorphisms. Eur. J. Hum. Genet. 27, 738–746. 10.1038/s41431-018-0292-2 30679813 PMC6462006

[B24] Kong S. W. Collins C. D. Shimizu-Motohashi Y. Holm I. A. Campbell M. G. Lee I.-H.. (2012). Characteristics and predictive value of blood transcriptome signature in males with autism spectrum disorders. PLoS ONE 7:e49475. 10.1371/journal.pone.0049475 23227143 PMC3515554

[B25] Krupp D. R. Barnard R. A. Duffourd Y. Evans S. A. Mulqueen R. M. Bernier R.. (2017). Exonic mosaic mutations contribute risk for autism spectrum disorder. Am. J. Hum. Genet. 101, 369–390. 10.1016/j.ajhg.2017.07.016 28867142 PMC5590950

[B26] Lin C.-Y. Chang K.-W. Lin C.-Y. Wu J.-Y. Coon H. Huang P.-H.. (2018). Allele-specific expression in a family quartet with autism reveals mono-to-Biallelic switch and novel transcriptional processes of autism susceptibility genes. Sci. Rep. 8, 1–15. 10.1038/s41598-018-22753-4 29523860 PMC5844893

[B27] Lippert C. Casale F. P. Rakitsch B. Stegle O. (2014). LIMIX: genetic analysis of multiple traits. BioRxiv. 10.1101/003905

[B28] Lord C. Rutter M. Goode S. Heemsbergen J. Jordan H. Mawhood L.. (1989). Austism diagnostic observation schedule: a standardized observation of communicative and social behavior. J. Autism Dev. Disord. 19, 185–212. 10.1007/BF02211841 2745388

[B29] Lord C. Rutter M. Le Couteur A. (1994). Autism diagnostic interview-revised: a revised version of a diagnostic interview for caregivers of individuals with possible pervasive developmental disorders. J. Autism Dev. Disord. 24, 659–685. 10.1007/BF02172145 7814313

[B30] Lovato D. V. Herai R. R. Pignatari G. C. Beltr ao-Braga P. C. (2019). The relevance of variants with unknown significance for autism spectrum disorder considering the genotype-phenotype interrelationship. Front. Psychiatry 10:409. 10.3389/fpsyt.2019.00409 31231258 PMC6567929

[B31] Martin J. Cooper M. Hamshere M. L. Pocklington A. Scherer S. W. Kent L.. (2014). Biological overlap of attention-deficit/hyperactivity disorder and autism spectrum disorder: evidence from copy number variants. J. Am. Acad. Child Adolesc. Psychiatry 53, 761–770. 10.1016/j.jaac.2014.03.004 24954825 PMC4074351

[B32] Matta J. Dobrino D. Howard S. Yeboah D. Kopel J. EL-Manzalawy Y.. (2021). A pheWAS model of autism spectrum disorder, in 2021 43rd Annual International Conference of the IEEE Engineering in Medicine & *Biology Society* (New York, NY: IEEE). 10.1109/EMBC46164.2021.9629533 34891705

[B33] Matta J. Obafemi-Ajayi T. Borwey J. Sinha K. Wunsch D. Ercal G. (2018a). Node-based resilience measure clustering with applications to noisy and overlapping communities in complex networks. Appl. Sci. 8:1307. 10.3390/app8081307

[B34] Matta J. Zhao J. Ercal G. Obafemi-Ajayi T. (2018b). Applications of node-based resilience graph theoretic framework to clustering autism spectrum disorders phenotypes. Appl. Netw. Sci. 3:38. 10.1007/s41109-018-0093-0 30839816 PMC6214326

[B35] Matta J. (2017). A comparison of approaches to computing betweenness centrality for large graphs, in International Conference on Complex Networks and Their Applications (Cham: Springer), 3–13. 10.1007/978-3-319-72150-7_1

[B36] Miles J. (2011). Autism subgroups from a medical genetics perspective, in Autism Spectrum Disorders, eds D. Amarel, D. Geschwind, and G. Dawson (New York, NY: Oxford University Press), 705–721. 10.1093/med/9780195371826.003.0046

[B37] Miles J. H. (2015). Complex autism spectrum disorders and cutting-edge molecular diagnostic tests. JAMA 314, 879–880. 10.1001/jama.2015.9577 26325555

[B38] Miles J. H. Takahashi T. N. Hong J. Munden N. Flournoy N. Braddock S. R.. (2008). Development and validation of a measure of dysmorphology: useful for autism subgroup classification. Am. J. Med. Genet. Part A 146, 1101–1116. 10.1002/ajmg.a.32244 18383511

[B39] Narita A. Nagai M. Mizuno S. Ogishima S. Tamiya G. Ueki M.. (2020). Clustering by phenotype and genome-wide association study in autism. Transl. Psychiatry 10, 1–12. 10.1038/s41398-020-00951-x 32807774 PMC7431539

[B40] Nguyen T. Nowell K. Bodner K. E. Obafemi-Ajayi T. (2018). Ensemble validation paradigm for intelligent data analysis in autism spectrum disorders, in 2018 IEEE Conference on Computational Intelligence in Bioinformatics and Computational Biology (New York, NY: IEEE), 1–8. 10.1109/CIBCB.2018.8404960

[B41] Poirier K. Hubert L. Viot G. Rio M. Billuart P. Besmond C.. (2017). CSNK2B splice site mutations in patients cause intellectual disability with or without myoclonic epilepsy. Hum. Mutat. 38, 932–941. 10.1002/humu.23270 28585349

[B42] Ruisch I. H. Dietrich A. Glennon J. C. Buitelaar J. K. Hoekstra P. J. (2019). Interplay between genome-wide implicated genetic variants and environmental factors related to childhood antisocial behavior in the UK Alspac cohort. Eur. Arch. Psychiatry Clin. Neurosci. 269, 741–752. 10.1007/s00406-018-0964-5 30569215 PMC6689282

[B43] Sanders A. R. Drigalenko E. I. Duan J. Moy W. Freda J. Göring H.. (2017). Transcriptome sequencing study implicates immune-related genes differentially expressed in schizophrenia: new data and a meta-analysis. Transl. Psychiatry 7:e1093. 10.1038/tp.2017.47 28418402 PMC5416689

[B44] Spencer M. Takahashi N. Chakraborty S. Miles J. Shyu C.-R. (2018). Heritable genotype contrast mining reveals novel gene associations specific to autism subgroups. J. Biomed. Inform. 77, 50–61. 10.1016/j.jbi.2017.11.016 29197649 PMC5788310

[B45] Stessman H. A. Xiong B. Coe B. P. Wang T. Hoekzema K. Fenckova M.. (2017). Targeted sequencing identifies 91 neurodevelopmental-disorder risk genes with autism and developmental-disability biases. Nat. Genet. 49, 515–526. 10.1038/ng.3792 28191889 PMC5374041

[B46] Tammimies K. Marshall C. R. Walker S. Kaur G. Thiruvahindrapuram B. Lionel A. C.. (2015). Molecular diagnostic yield of chromosomal microarray analysis and whole-exome sequencing in children with autism spectrum disorder. JAMA 314, 895–903. 10.1001/jama.2015.10078 26325558

[B47] Traag V. Waltman L. van Eck N. J. (2018). From Louvain to Leiden: guaranteeing well-connected communities. arXiv preprint arXiv:1810.08473. 10.1038/s41598-019-41695-z 30914743 PMC6435756

[B48] Tyler A. L. Crawford D. C. Pendergrass S. A. (2016). The detection and characterization of pleiotropy: discovery, progress, and promise. Brief. Bioinformatics 17, 13–22. 10.1093/bib/bbv050 26223525

[B49] Verma A. Bang L. Miller J. E. Zhang Y. Lee M. T. M. Zhang Y.. (2019). Human-disease phenotype map derived from pheWAS across 38,682 individuals. Am. J. Hum. Genet. 104, 55–64. 10.1016/j.ajhg.2018.11.006 30598166 PMC6323551

[B50] Vevera J. Zarrei M. Hartmannová H. Jedličková I. Mušálková D. Přistoupilová A.. (2019). Rare copy number variation in extremely impulsively violent males. Genes Brain Behav. 18:e12536. 10.1111/gbb.12536 30411505

[B51] Vezain M. Lecuyer M. Rubio M. Dupé V. Ratié L. David V.. (2018). A de novo variant in ADGRL2 suggests a novel mechanism underlying the previously undescribed association of extreme microcephaly with severely reduced sulcation and rhombencephalosynapsis. Acta Neuropathol. Commun. 6, 1–23. 10.1186/s40478-018-0610-5 30340542 PMC6195752

[B52] Vorstman J. A. Van Daalen E. Jalali G. R. Schmidt E. R. Pasterkamp R. J. de Jonge M.. (2011). A double hit implicates DIAPH3 as an autism risk gene. Mol. Psychiatry 16, 442–451. 10.1038/mp.2010.26 20308993 PMC3334288

[B53] Wang J. Qiao J.-D. Liu X.-R. Liu D.-T. Chen Y.-H. Wu Y.. (2021). Unc13b variants associated with partial epilepsy with favourable outcome. Brain 144, 3050–3060. 10.1093/brain/awab164 33876820 PMC8634081

[B54] Williams S. M. An J. Y. Edson J. Watts M. Murigneux V. Whitehouse A. J.. (2019). An integrative analysis of non-coding regulatory dna variations associated with autism spectrum disorder. Mol. Psychiatry 24, 1707–1719. 10.1038/s41380-018-0049-x 29703944

[B55] Wu H. Li H. Bai T. Han L. Ou J. Xun G.. (2020). Phenotype-to-genotype approach reveals head-circumference-associated genes in an autism spectrum disorder cohort. Clin. Genet. 97, 338–346. 10.1111/cge.13665 31674007 PMC7307605

[B56] Xue C.-B. Xu Z.-H. Zhu J. Wu Y. Zhuang X.-H. Chen Q.-L.. (2019). Exome sequencing identifies TENM4 as a novel candidate gene for schizophrenia in the SCZD2 Locus at 11q14-21. Front. Genet. 9:725. 10.3389/fgene.2018.00725 30745909 PMC6360184

[B57] Yang J. Leskovec J. (2015). Defining and evaluating network communities based on ground-truth. Knowl. Inform. Syst. 42, 181–213. 10.1007/s10115-013-0693-z

[B58] Yang C.-H. Lin Y.-D. Chuang L.-Y. (2018). Class balanced multifactor dimensionality reduction to detect gene-gene interactions. IEEE/ACM Trans. Comput. Biol. Bioinformatics. 17, 71–81. 10.1109/TCBB.2018.2858776 30040653

[B59] Zhao Y. Liang X. Zhu F. Wen Y. Xu J. Yang J.. (2018). A large-scale integrative analysis of GWAS and common meQTLs across whole life course identifies genes, pathways and tissue/cell types for three major psychiatric disorders. Neurosci. Biobehav. Rev. 95, 347–352. 10.1016/j.neubiorev.2018.10.005 30339835

[B60] Zhao J. Nguyen T. Kopel J. Koob P. B. Adjeroh D. A. Obafemi-Ajayi T. (2019). Genotype combinations linked to phenotype subgroups in autism spectrum disorders, in 2019 IEEE Conference on Computational Intelligence in Bioinformatics and Computational Biology (New York, NY: IEEE), 1–8. 10.1109/CIBCB.2019.8791461

